# Supplementary figures and images for: Multi-scale and multi-site resampling of a study area in spatial genetics: implications for flying insect species
Source: PeerJ. 2017 Dec 15;5:e4135. doi: 10.7717/peerj.4135 (PMC5733902; doi:10.7717/peerj.4135)

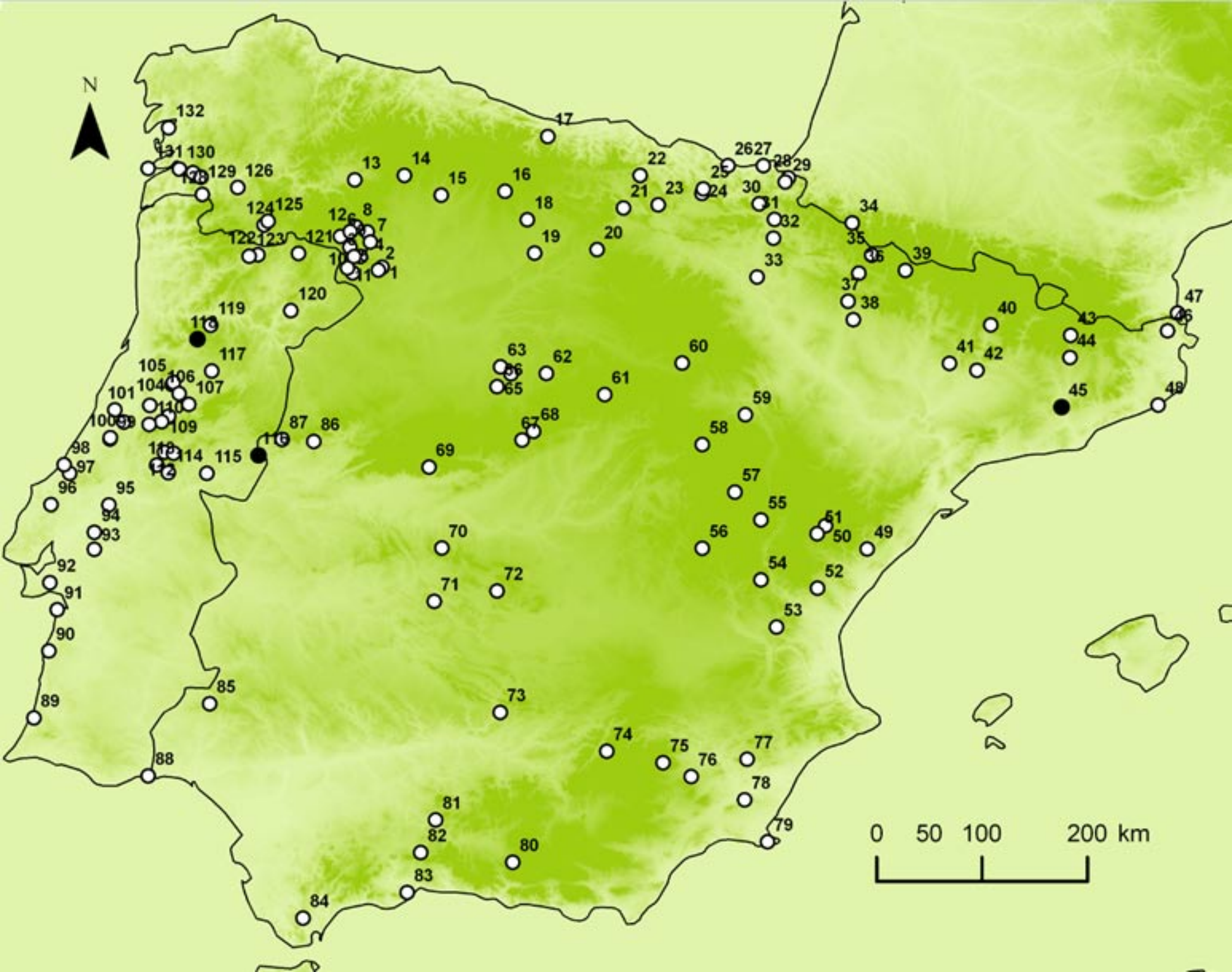

Supplement: Figure S1 — Black dots refer to populations of size >19 individuals. The green background refers to elevation (from pale to dark green: low to high elevation). [file peerj-05-4135-s001.pdf]

Mean number of individuals in sampling areas

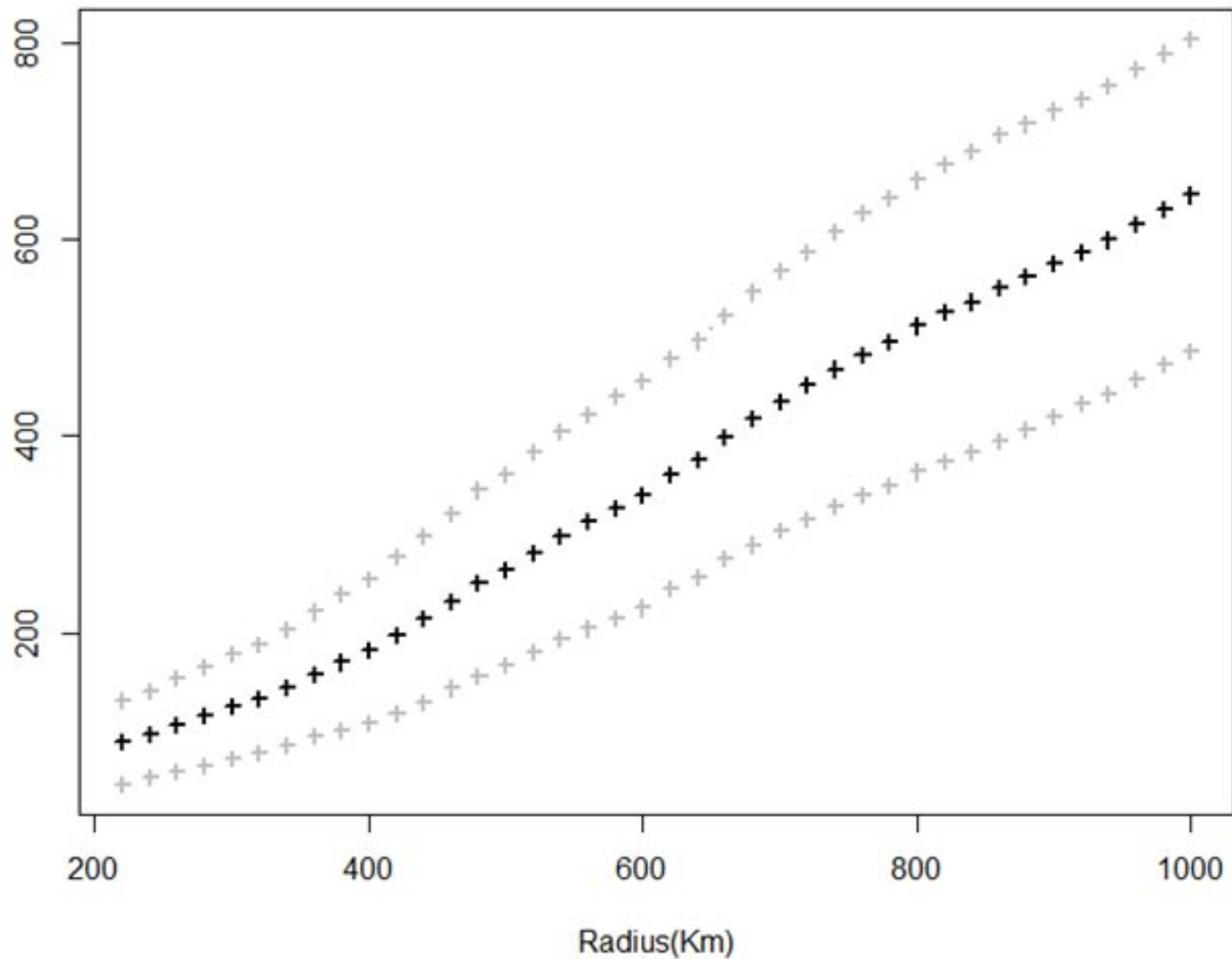

Supplement: Figure S2 — Mean: black; ±SD: grey [file peerj-05-4135-s002.pdf]

$$\text{DeltaK} = \text{mean}(|L''(K)|) / \text{sd}(L(K))$$

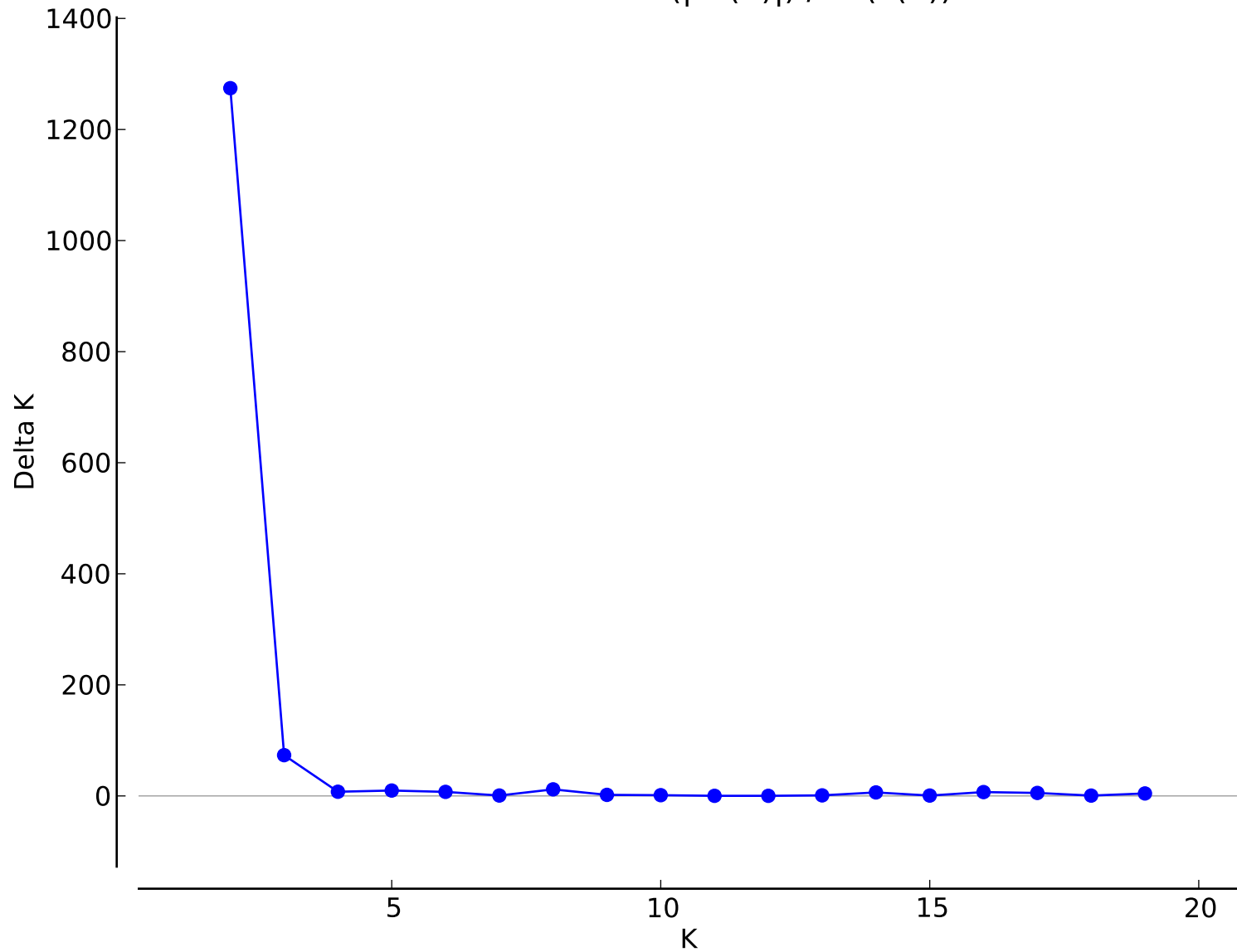

Supplement: Figure S3 [file peerj-05-4135-s003.pdf]
