# Supplementary material for: Multi-scale and multi-site resampling of a study area in spatial genetics: implications for flying insect species
Source: PeerJ. 2017 Dec 15;5:e4135. doi: 10.7717/peerj.4135 (PMC5733902; doi:10.7717/peerj.4135)
Supplement: Table S1 — Long. and Lat. refer to the geographic coordinates of the sampling sites; N, is the number of individuals of demes; A, mean allelic richness; AR, corrected allelic richness, accounting for variation in deme size; Fis, Fis estimate of deme, computed without Mon01 and Mon 27. [file peerj-05-4135-s004.docx]

| Long. and Lat. refer to geographic coordinates of sampling sites; N. is the number of individuals of demes; A. mean allelic richness; AR. corrected allelic richness, accounting to variation in deme size; *F_is_*. *F_is_* estimate of deme, computed without Mon01 and Mon 27 | | | | | | |  |  |  |
| --- | --- | --- | --- | --- | --- | --- | --- | --- | --- |
| **Code** | **Locality** | **Country** | **Year** | **Long.** | **Lat.** | **N.** | **A** | **AR** | ***Fis*** |
| **Eastern Iberian lineage** | |  |  |  |  |  |  |  |  |
| 1 | Zamora Tabaras | Spain | 2012 | -6,04 | 41,87 | 10 | 3,23 | 1,55 | 0,11 |
| 2 | Zamora ferreras | Spain | 2012 | -6,08 | 41,84 | 10 | 2,85 | 1,51 | 0,20 |
| 3 | Zamora Villar | Spain | 2012 | -6,31 | 41,92 | 10 | 3,38 | 1,56 | 0,24 |
| 4 | Justel | Spain | 2012 | -6,29 | 42,14 | 8 | 3,85 | 1,64 | 0,14 |
| 5 | San Vitero | Spain | 2012 | -6,36 | 41,77 | 10 | 3,62 | 1,56 | 0,31 |
| 6 | Puebla De Sanabria | Spain | 2012 | -6,58 | 42,05 | 8 | 3,31 | 1,60 | 0,17 |
| 7 | Manzanal Infantes | Spain | 2012 | -6,23 | 42,06 | 5 | 3,38 | 1,58 | 0,13 |
| 8 | Espadanedo | Spain | 2012 | -6,43 | 42,16 | 2 | _ | _ | _ |
| 9 | Manzana De Arriba | Spain | 2012 | -6,45 | 41,98 | 9 | 3,38 | 1,58 | 0,14 |
| 10 | Mahide | Spain | 2012 | -6,38 | 41,91 | 5 | 2,92 | 1,57 | 0,29 |
| 11 | Figueruela De Arriba | Spain | 2012 | -6,43 | 41,80 | 5 | 3,00 | 1,53 | -0,07 |
| 12 | Rosinos | Spain | 2012 | -6,48 | 42,11 | 10 | 3,31 | 1,57 | 0,03 |
| 13 | Ponferrada | Spain | 2012 | -6,55 | 42,55 | 12 | 3,54 | 1,49 | -0,04 |
| 14 | Quintana de Castillo | Spain | 2012 | -6,00 | 42,68 | 10 | 3,92 | 1,58 | 0,05 |
| 15 | Leon | Spain | 2013 | -5,54 | 42,58 | 3 | _ | _ | _ |
| 16 | Villalba de Guardo | Spain | 2012 | -4,82 | 42,72 | 15 | 3,31 | 1,49 | 0,15 |
| 17 | Lamason | Spain | 2012 | -4,45 | 43,25 | 7 | 2,85 | 1,54 | 0,06 |
| 18 | Villanúno de Valdavia | Spain | 2012 | -4,51 | 42,52 | 10 | 3,31 | 1,49 | 0,12 |
| 19 | Cuellar | Spain | 2012 | -4,36 | 42,25 | 6 | 3,31 | 1,60 | 0,08 |
| 20 | Burgos | Spain | 2013 | -3,66 | 42,38 | 2 | _ | _ | _ |
| 21 | Ona | Spain | 2012 | -3,44 | 42,77 | 9 | 3,62 | 1,55 | 0,09 |
| 22 | Villanueva de Mena | Spain | 2012 | -3,31 | 43,07 | 5 | 2,92 | 1,51 | 0,31 |
| 23 | Espejo | Spain | 2012 | -3,05 | 42,85 | 3 | _ | _ | _ |
| 24 | Vitoria | Spain | 2013 | -2,57 | 43,01 | 7 | 2,85 | 1,46 | 0,21 |
| 25 | Aramayona | Spain | 2012 | -2,56 | 43,05 | 5 | 2,23 | 1,45 | 0,26 |
| 26 | Itziar | Spain | 2013 | -2,32 | 43,28 | 14 | 3,38 | 1,49 | 0,20 |
| 27 | Irun | Spain | 2013 | -1,91 | 43,33 | 18 | 3,46 | 1,47 | 0,19 |
| 28 | Sare | France | 2013 | -1,60 | 43,26 | 18 | 3,31 | 1,48 | 0,31 |
| 29 | Etchalar | Spain | 2013 | -1,63 | 43,22 | 10 | 3,00 | 1,49 | 0,38 |
| 30 | Lekunberri | Spain | 2012 | -1,89 | 43,00 | 2 | _ | _ | _ |
| 31 | Marcalain | Spain | 2012 | -1,69 | 42,89 | 11 | 3,62 | 1,53 | 0,19 |
| 32 | Beriain | Spain | 2012 | -1,67 | 42,73 | 10 | 2,62 | 1,47 | 0,14 |
| 33 | Falces | Spain | 2012 | -1,79 | 42,38 | 14 | 4,31 | 1,54 | 0,18 |
| 34 | Col St Martin | Spain | 2013 | -0,79 | 42,96 | 10 | 3,77 | 1,57 | 0,15 |
| 35 | Canfranc | Spain | 2013 | -0,52 | 42,71 | 17 | 4,77 | 1,60 | 0,06 |
| 36 | Jaca | Spain | 2013 | -0,64 | 42,54 | 16 | 4,38 | 1,56 | 0,11 |
| 37 | Huesca 1 | Spain | 2013 | -0,72 | 42,29 | 18 | 4,23 | 1,57 | 0,16 |
| 38 | Huesca 2 | Spain | 2013 | -0,63 | 42,14 | 7 | 3,23 | 1,51 | 0,28 |
| 39 | Torla | Spain | 2012 | -0,11 | 42,62 | 1 | _ | _ | _ |
| 40 | La Pobla De Segur | Spain | 2013 | 0,94 | 42,25 | 18 | 4,38 | 1,60 | 0,15 |
| 41 | Castillonroy | Spain | 2013 | 0,52 | 41,88 | 10 | 3,69 | 1,54 | 0,11 |
| 42 | Camarassa | Spain | 2013 | 0,84 | 41,85 | 6 | 2,92 | 1,55 | 0,22 |
| 43 | Baga | Spain | 2013 | 1,86 | 42,24 | 18 | 3,92 | 1,55 | 0,08 |
| 44 | Olvan | Spain | 2013 | 1,88 | 42,05 | 18 | 4,00 | 1,52 | 0,06 |
| 45 | Castellbell | Spain | 2013 | 1,84 | 41,62 | 30 | 4,46 | 1,60 | 0,14 |
| 46 | Capmany | Spain | 2014 | 2,96 | 42,36 | 11 | 3,15 | 1,59 | 0,19 |
| 47 | Argelès | France | 2013 | 3,06 | 42,52 | 10 | 3,15 | 1,51 | 0,11 |
| 48 | Tossa de Mar | Spain | 2012 | 2,93 | 41,72 | 10 | 3,38 | 1,52 | 0,10 |
| 49 | Atzeneta | Spain | 2012 | -0,15 | 40,21 | 10 | 3,92 | 1,60 | 0,09 |
| 50 | Alcala | Spain | 2012 | -0,64 | 40,36 | 7 | 3,38 | 1,58 | 0,04 |
| 51 | Mora | Spain | 2012 | -0,72 | 40,28 | 10 | 3,85 | 1,59 | 0,15 |
| 52 | Cuenca Altura | Spain | 2012 | -0,64 | 39,82 | 10 | 3,69 | 1,56 | 0,08 |
| 53 | Requena | Spain | 2012 | -1,03 | 39,44 | 9 | 3,85 | 1,62 | 0,02 |
| 54 | Cuenca Talayuelas | Spain | 2012 | -1,27 | 39,82 | 10 | 3,23 | 1,51 | 0,05 |
| 55 | Albaracin | Spain | 2012 | -1,36 | 40,33 | 9 | 3,92 | 1,57 | -0,01 |
| 56 | Cuenca Palancares | Spain | 2012 | -1,96 | 40,01 | 8 | 3,46 | 1,57 | 0,13 |
| 57 | Oribuela | Spain | 2012 | -1,69 | 40,53 | 1 | _ | _ | _ |
| 58 | Cobeta | Spain | 2012 | -2,13 | 40,89 | 12 | 3,85 | 1,59 | 0,02 |
| 59 | Muneberga | Spain | 2012 | -1,70 | 41,20 | 10 | 4,08 | 1,56 | 0,22 |
| 60 | Almazan | Spain | 2012 | -2,49 | 41,55 | 7 | 3,62 | 1,57 | 0,18 |
| 61 | Catalojas | Spain | 2012 | -3,30 | 41,17 | 8 | 3,54 | 1,55 | -0,01 |
| 62 | Catalejo | Spain | 2012 | -3,99 | 41,26 | 10 | 3,62 | 1,55 | 0,31 |
| 63 | Segovia Coca | Spain | 2012 | -4,51 | 41,24 | 10 | 3,62 | 1,51 | 0,09 |
| 64 | Segovia Navas | Spain | 2012 | -4,38 | 41,20 | 10 | 3,62 | 1,51 | 0,23 |
| 65 | Segovia Santa Maria | Spain | 2012 | -4,51 | 41,07 | 9 | 4,15 | 1,60 | 0,08 |
| 66 | Santa Maria | Spain | 2012 | -4,51 | 41,08 | 10 | 4,31 | 1,59 | 0,09 |
| 67 | Guadarrama | Spain | 2012 | -4,13 | 40,66 | 4 | _ | _ | _ |
| 68 | Cercedilla | Spain | 2012 | -4,02 | 40,75 | 12 | 4,00 | 1,58 | 0,07 |
| 69 | El Arenal | Spain | 2012 | -5,10 | 40,28 | 12 | 4,54 | 1,64 | 0,11 |
| 70 | Espinoso Del Rey | Spain | 2012 | -4,80 | 39,62 | 10 | 4,08 | 1,57 | 0,12 |
| 71 | Puebla De Don Rodrigo | Spain | 2012 | -4,78 | 39,16 | 7 | 3,08 | 1,53 | 0,27 |
| 72 | Los Yebenes | Spain | 2012 | -4,12 | 39,35 | 1 | _ | _ | _ |
| 73 | El Centimillo | Spain | 2012 | -3,87 | 38,33 | 1 | _ | _ | _ |
| 74 | Santiago Murcia | Spain | 2012 | -2,67 | 38,16 | 10 | 3,85 | 1,54 | 0,13 |
| 75 | Murcia Bebedor | Spain | 2012 | -2,05 | 38,14 | 10 | 3,46 | 1,55 | 0,14 |
| 76 | Cehegin | Spain | 2012 | -1,73 | 38,06 | 3 | _ | _ | _ |
| 77 | Murcia Puerto | Spain | 2012 | -1,16 | 38,28 | 9 | 3,62 | 1,53 | 0,17 |
| 78 | Murcia Valle | Spain | 2012 | -1,13 | 37,93 | 4 | _ | _ | _ |
| 79 | Huerta-Cartagena | Spain | 2012 | -0,83 | 37,60 | 1 | _ | _ | _ |
| 80 | Monachil | Spain | 2012 | -3,48 | 37,08 | 7 | 3,46 | 1,54 | 0,07 |
| 81 | Rute | Spain | 2012 | -4,36 | 37,32 | 9 | 3,62 | 1,56 | 0,10 |
| 82 | Antequera | Spain | 2012 | -4,46 | 37,02 | 2 | _ | _ | _ |
| 83 | El Pastor | Spain | 2012 | -4,53 | 36,66 | 8 | 2,77 | 1,48 | 0,23 |
| 84 | LosBarrios | Spain | 2012 | -5,56 | 36,27 | 6 | 2,77 | 1,52 | 0,28 |
| 85 | Aroche | Spain | 2012 | -6,96 | 37,90 | 11 | 3,38 | 1,41 | -0,05 |
| 86 | Ovejuela | Spain | 2012 | -6,41 | 40,29 | 10 | 3,31 | 1,50 | 0,24 |
| 87 | El Payo | Spain | 2012 | -6,76 | 40,25 | 10 | 3,46 | 1,46 | 0,09 |
|  |  |  |  |  |  |  |  |  |  |
| **Western Iberian lineage** | |  |  |  |  |  |  |  |  |
| 88 | Monte Gordo | Portugal | 2013 | -7,45 | 37,18 | 12 | 2,46 | 1,43 | -0,06 |
| 89 | Odeceixe | Portugal | 2013 | -8,77 | 37,43 | 8 | 2,15 | 1,32 | -0,03 |
| 90 | Santo Andres | Portugal | 2013 | -8,77 | 38,02 | 12 | 2,31 | 1,31 | 0,19 |
| 91 | Comporta | Portugal | 2011 | -8,78 | 38,38 | 4 | _ | _ | _ |
| 92 | Palmela | Portugal | 2013 | -8,92 | 38,59 | 1 | _ | _ | _ |
| 93 | Coruche | Portugal | 2011 | -8,53 | 38,96 | 3 | _ | _ | _ |
| 94 | Santarem | Portugal | 2013 | -8,57 | 39,10 | 10 | 2,92 | 1,49 | 0,08 |
| 95 | Chamusca | Portugal | 2011 | -8,48 | 39,36 | 3 | _ | _ | _ |
| 96 | Cadaval | Portugal | 2011 | -9,10 | 39,24 | 3 | _ | _ | _ |
| 97 | Alcobaça | Portugal | 2011 | -8,98 | 39,54 | 4 | _ | _ | _ |
| 98 | Nazaré | Portugal | 2011 | -9,06 | 39,60 | 1 | _ | _ | _ |
| 99 | Pombal | Portugal | 2011 | -8,63 | 39,91 | 1 | _ | _ | _ |
| 100 | Pombal | Portugal | 2011 | -8,63 | 39,92 | 2 | _ | _ | _ |
| 101 | Montemor | Portugal | 2011 | -8,65 | 40,16 | 2 | _ | _ | _ |
| 102 | Coimbra | Portugal | 2013 | -8,52 | 40,08 | 12 | 2,54 | 1,38 | 0,01 |
| 103 | Penacova | Portugal | 2011 | -8,28 | 40,27 | 1 | _ | _ | _ |
| 104 | Tondela | Portugal | 2013 | -8,09 | 40,49 | 12 | 2,92 | 1,45 | -0,10 |
| 105 | Tondela 2 | Portugal | 2011 | -8,08 | 40,51 | 2 | _ | _ | _ |
| 106 | Carregal do sal | Portugal | 2011 | -7,99 | 40,43 | 3 | _ | _ | _ |
| 107 | Oliviera do hospital | Portugal | 2011 | -7,86 | 40,36 | 1 | _ | _ | _ |
| 108 | Arganil PT | Portugal | 2011 | -8,05 | 40,22 | 3 | _ | _ | _ |
| 109 | Gois | Portugal | 2011 | -8,11 | 40,16 | 4 | _ | _ | _ |
| 110 | Lousa | Portugal | 2011 | -8,24 | 40,11 | 1 | _ | _ | _ |
| 111 | Alvelos | Portugal | 2011 | -8,01 | 39,91 | 2 | _ | _ | _ |
| 112 | Oleiros | Portugal | 2011 | -7,91 | 39,92 | 5 | 2,23 | 1,38 | -0,27 |
| 113 | Serta | Portugal | 2011 | -8,06 | 39,79 | 1 | _ | _ | _ |
| 114 | Proenca A Nova | Portugal | 2011 | -7,92 | 39,75 | 1 | _ | _ | _ |
| 115 | Castelo branco | Portugal | 2011 | -7,50 | 39,82 | 4 | _ | _ | _ |
| 116 | Vale Feitoso | Portugal | 2011 | -6,98 | 40,07 | 19 | 3,38 | 1,46 | 0,15 |
| 117 | Penlava Do Castelo | Portugal | 2011 | -7,69 | 40,68 | 1 | _ | _ | _ |
| 118 | Castro Daire | Portugal | 2013 | -7,92 | 40,92 | 24 | 3,77 | 1,52 | 0,03 |
| 119 | Lamego | Portugal | 2013 | -7,81 | 41,06 | 6 | 2,54 | 1,50 | 0,08 |
| 120 | Alfandega Da Fe | Portugal | 2011 | -6,96 | 41,34 | 3 | _ | _ | _ |
| 121 | Vinhais | Portugal | 2011 | -7,01 | 41,83 | 2 | _ | _ | _ |
| 122 | Chaves | Portugal | 2011 | -7,46 | 41,74 | 1 | _ | _ | _ |
| 123 | Redondelo | Portugal | 2013 | -7,55 | 41,71 | 12 | 3,23 | 1,47 | 0,03 |
| 124 | Vences | Spain | 2011 | -7,46 | 42,00 | 2 | _ | _ | _ |
| 125 | Matama | Spain | 2011 | -7,43 | 42,04 | 8 | 3,00 | 1,52 | -0,02 |
| 126 | Ourense | Spain | 2013 | -7,85 | 42,26 | 12 | 3,23 | 1,45 | 0,11 |
| 127 | Ventin | Portugal | 2011 | -8,39 | 42,29 | 1 | _ | _ | _ |
| 128 | Ribeira | Spain | 2011 | -8,23 | 42,13 | 1 | _ | _ | _ |
| 129 | Riva Crecente | Spain | 2011 | -8,29 | 42,27 | 1 | _ | _ | _ |
| 130 | Amoedos | Spain | 2011 | -8,55 | 42,29 | 1 | _ | _ | _ |
| 131 | VigaIslas | Spain | 2011 | -8,90 | 42,23 | 3 | _ | _ | _ |
| 132 | Villagarcia de Aroussa | Spain | 2011 | -8,78 | 42,61 | 9 | 3,00 | 1,47 | 0,08 |
|  |  |  |  |  |  |  |  |  |  |
| **Average Eastern Iberian lineage** | |  |  |  |  |  |  |  |  |
| _ | _ | _ | _ | _ | **_** | 9,08 | 3,51 | 1,54 | 0,19 |
| **Average Western Iberian lineage** | | |  |  |  |  |  |  |  |
| _ | _ | _ | _ | _ | **_** | 4,98 | 2,84 | 1,44 | 0,1 |
| **Average Iberian peninsula** | |  |  |  |  |  |  |  |  |
| _ | _ | _ | _ | _ | **_** | 7,68 | 3,43 | 1,53 | 0,22 |
