# Supplementary material for: Multi-scale and multi-site resampling of a study area in spatial genetics: implications for flying insect species
Source: PeerJ. 2017 Dec 15;5:e4135. doi: 10.7717/peerj.4135 (PMC5733902; doi:10.7717/peerj.4135)
Supplement: Table S2 — Multiplexed PCR were performed in a 10 µL reaction volume using 25 ng of genomic DNA, 0.4 U of DreamTaq DNA Polymerase (Thermo Scientific®), 0.75 µL Dream Taq Green Buffer (including 20 mM MgCl2, Thermo Scientific®), 1 µM Betaine, 0.24 µL dNTP (10 µM) and deionized H2O. PCR amplifications were run on a Veriti® 96 well fast thermal cycler (Applied Biosystems®) using the following settings: a first denaturation step at 95 °C for 10 min; 40 cycles of denaturation (30 s at 95 °C), hybridization (30 s at 55 °C) and elongation (1 min at 72 °C), and a final elongation step at 72 °C for 10 min. One µL of PCR products was denatured in a mix of 10 µL of formamide and 0.3 µL of 600 Liz marker before being run on an ABI PRISM 3500 sequencer (Life Technologies®). Genotypes were read using GENEMAPPER V 4.1 software (Applied Biosystems®). [file peerj-05-4135-s005.docx]

| **Locus** | **Primers sequence 5' -> 3'** | **Multiplex** | **Amount (pmol)** | **Motif** | **Size range** |
| --- | --- | --- | --- | --- | --- |
| Mon 01 | F: TTCACGCACATCATTTCTTTG | 1 | 2.4 | (aac)5 | 122-146 |
|  | R: TCAAGCAGGAAACGAAAAGC |  |  |  |  |
| Mon 08 | F: TGGTGTCTGTAGAACGCTTCA | 3 | 1 | (tatc)5 | 192-196 |
|  | R: GCTTATTAGCTCTCATCAGTATGCTC |  |  |  |  |
| Mon 17 | F: TAGTTTTACTGGGGCCAATG | 3 | 1.6 | (gt)6 | 149-153 |
|  | R: GAACTCATGAACGGATATAAATGAA |  |  |  |  |
| Mon 23 | F: ATTTATTCCAAATTGCCAATACTACA | 1 | 2 | (ca)7 | 142-144 |
|  | R: GTGTAAGGTGGAAGTGTCAAAGC |  |  |  |  |
| Mon 27 | F: ACAATCTCTTTCGATACCGTTGA | 3 | 2 | (tg)7 | 118-124 |
|  | R: TTTGCTACAAAGATGTTCTTAAAAGT |  |  |  |  |
| Mon 30 | F: TTTCTAGTTTGCCTGTATCCCG | 1 | 1.6 | (ag)8 | 236-248 |
|  | R: AAAGCGGGTGTGAAGTACCA |  |  |  |  |
| Mon 31 | F: GTAAGAGAACCCAACCACCG | 2 | 0.64 | (ag)8 | 146-150 |
|  | R: TTATCCTCACCGGACCGTTA |  |  |  |  |
| Mon 35 | F: TTTCATCTGACTTAATTTTCTTTCTCA | 2 | 7.2 | (tc)8 | 110-126 |
|  | R: AGGGACGTGCAGATTAGGAA |  |  |  |  |
| Mon 36 | F: ACGGTGCAGAACTAAAGTTAGCC | 2 | 0.8 | (tg)8 | 196-200 |
|  | R: GACTCGGACGGAGCTTCT |  |  |  |  |
| Mon 41 | F: ACGGTAGCGCAATCTTGAGT | 3 | 0.8 | (ct)9 | 116-120 |
|  | R: ACGTGAGCAGTCCTGTTGC |  |  |  |  |
| Mon 42 | F: CCTTATTTAGGTCAAGAATTCGC | 1 | 1.6 | (gt)9 | 138-146 |
|  | R: GCGTGTCATTATTCCAAGGAC |  |  |  |  |
| Mon 44 | F: AACCTGGACCTAGCTCGGAA | 2 | 0.64 | (act)10 | 98-113 |
|  | R: TAGGAGAAGGTGGAGCAGGA |  |  |  |  |
|  |  |  |  |  |  |
